# Introduction to the special section on cancer survivors and treatment-related accelerated aging

**DOI:** 10.1007/s11764-024-01613-3

**Published:** 2024-05-15

**Authors:** Lisa Gallicchio, Jennifer L. Guida, Paige A. Green

**Affiliations:** 1 Epidemiology and Genomics Research Program, Division of Cancer Control and Population Sciences, National Cancer Institute, 9609 Medical Center Drive, Rockville, MD 20850 USA; 2 Behavioral Research Program, Division of Cancer Control and Population Sciences, National Cancer Institute, Rockville, MD USA

**Keywords:** Accelerated aging, Biomarkers, Cognitive functioning, Cancer survivors, Frailty, Physical functioning

Contemporary advances in cancer treatment have dramatically increased cancer survival rates over the past several decades, leading to an estimated 18.1 million cancer survivors living in the United States [1, 2]. Yet, such treatments have been shown in laboratory studies to cause cell damage through many of the same mechanisms that are thought to underlie the normal aging process [3]. Thus, the treatments that spare cancer survivors from mortality related to their cancer may adversely alter their aging trajectory, putting the cancer survivor at risk for a broad spectrum of aging-related health conditions, including cognitive dysfunction, cardiovascular disease, second cancers, and frailty, at a younger age than would occur with the normal aging process [3, 4]. This phenomenon, known as “premature aging” or “accelerated aging,” can lead to decreased quality of life, reduced healthspan, and, ultimately, premature mortality for the cancer survivor [4].

This special section features studies examining trajectories of accelerated aging among cancer survivors, promising biomarkers to identify cancer survivors at risk of accelerated aging, and intervention strategies to prevent, mitigate, and reduce the aging effects of cancer and its treatment. Opportunities for future research directions are also highlighted. The goal of this special section is to increase awareness of the impact that cancer and its treatment have on the normal aging process, especially among clinicians who treat individuals with a history of cancer and may not be aware of adverse long-term outcomes that appear to be part of the normal aging process but are experienced earlier in life.

The first article of the special section is a scoping review of the literature published since 2018 conducted by Mohamed et al. [5] evaluating physical and cognitive functional outcomes in cancer survivors treated with chemotherapy. In 2018, the National Cancer Institute (NCI) held the first of two think tanks on the topic of cancer and accelerated aging, entitled “Measuring Aging and Identifying Aging Phenotypes in Cancer Survivors” [6]. The objectives of this first think tank were to identify aging phenotypes and trajectories experienced by cancer survivors and to assess current knowledge on the measurement of aging phenotypes and trajectories in this population. The purpose of holding the NCI-led think tank was to understand the state of the science, encourage the research community to begin generating evidence to fill gaps in knowledge, and identify future research directions [6]. In the Mohamed et al. [5] scoping review, the authors identified 65 observational studies published after the release of the 2018 NCI think tank report [7] that included cancer survivors, focused on the concepts of physical function, cognitive function, and/or frailty as outcomes, and reported on these outcomes in relation to chemotherapy treatment. The authors reported that the majority of the studies showed that chemotherapy is associated with reduced physical function, reduced cognitive function, and an increase in frailty among cancer survivors; these associations were observed across cancer survivor populations, including adult survivors of childhood cancer, as well as older cancer survivors. The review highlights the need to conduct longitudinal studies that evaluate aging-related outcomes over extended periods, including from diagnosis to years post-treatment, and that integrate assessments over multiple time points (e.g., pre-treatment, during treatment, post-treatment, and at several follow-ups) to enable examination of aging trajectories.

In an analysis of data from the Hurria Older PatiEnts (HOPE) with Breast Cancer Study, Sedrak et al. [8] examined the impact of adjuvant chemotherapy on functional decline in older women with early stage breast cancer. Notably, unlike many previously conducted studies in the published literature, the Sedrak et al. [8] study included control groups of age-matched women with early stage breast cancer who did not receive chemotherapy and age-matched women without a history of cancer. As seen in several of the articles identified in the Mohamed et al. [5] review, Sedrak et al. [8] showed that receipt of chemotherapy was associated with accelerated physical function decline among women with breast cancer, underscoring the need for the development of interventions to preserve physical function and improve health outcomes in this population.

Westrick et al. [9] utilized data from the Health and Retirement Study (HRS), a population-based longitudinal cohort study of older adults in the United States, to examine how cardiovascular risk factors (CVRF) present at the time of a cancer diagnosis impact subsequent memory aging post-diagnosis, and whether this relationship differs by race/ethnicity. Similar to Sedrak et al. [8], the Westrick et al. [9] study included a cancer-free control group, so the longitudinal associations between CVRF and memory aging among cancer survivors could be compared to those without a history of cancer. Interestingly, the authors found that a higher number of CVRFs at diagnosis were associated with worse memory over time; however, the trajectories did not differ between those with and without cancer. Non-Hispanic Black and Hispanic participants in the HRS displayed worse memory recall compared to non-Hispanic whites. However, the magnitude of these differences was similar between those with and without cancer. To our knowledge, this is one of the first studies to examine racial differences in aging trajectories among cancer survivors.

The use of biomarkers to (1) identify cancer survivors who are at risk for accelerated aging and (2) measure biological aging among cancer survivors to inform fit for treatment is an emerging area of interest. In a cross-sectional study of 1720 adult survivors of childhood cancer participating in the St. Jude Lifetime Cohort, Berkman et al. [10] reported that a higher peripheral blood mitochondrial DNA copy number (mtDNA-CN), a proxy for mitochondrial function, was significantly and inversely associated with global longitudinal strain, suggesting that mtDNA has a potential role as a biomarker for early cardiac dysfunction in this cancer survivor population. In contrast, no statistically significant associations were observed between mtDNA-CN and either exercise intolerance or walking inefficiency. In a longitudinal pilot study of older adults with hematologic malignancies, Rosko et al. [11] investigated the ability of multiple aging-related biomarkers (including p16 and several epigenetic clocks) to identify frailty, as measured by a newly developed, but not yet validated, clinical impairment index, prior to treatment. The authors showed that p16, a marker of cellular senescence, and three epigenetic clocks (the Hannum clock, the PhenoAge clock, and DunedinPACE) showed the greatest potential to identify frailty, suggesting that the measurement of these aging biomarkers prior to treatment could be utilized in place of a physical functioning assessment, such as the geriatric assessment [12], to predict treatment tolerance and tailor therapeutic strategies.

Finally, Winters-Stone et al. [13] conducted a secondary analysis of data from a randomized controlled trial to investigate whether tai ji quan or strength training was effective in improving an individual’s frailty phenotype (i.e., moving from a frail state to pre-frail/robust, or a pre-frail state to robust) or any component of the frailty phenotype (fatigue/exhaustion, weakness, slowness, inactivity) over 6 months among postmenopausal women with cancer. Findings showed that strength training was superior at improving an individual’s overall frailty phenotype compared to tai ji quan and the stretching control group. However, both strength training and tai ji quan favorably impacted the number of frailty criteria, as the participants in both of these groups were twice as likely to improve in at least one frailty criteria compared to stretching. These findings provide promise that there are feasible strategies to reduce, mitigate, and even reverse the adverse aging effects of cancer and its treatment among cancer survivors of middle to older ages.

The intention of this special section of the *Journal of Cancer Survivorship* was to highlight the importance of understanding the effects of cancer and its treatment on aging phenotypes and trajectories, identifying the cancer survivors who are most at risk of experiencing accelerated aging, and generating strategies to reduce or mitigate the adverse aging-related effects of cancer and its treatment. Numerous opportunities for future research continue to exist in this scientific area, including many noted in the published reports from 2018 and 2019 NCI think tanks [6, 14]. Indeed, many gaps in knowledge remain.

First, there is a continued need to identify the specific demographic, clinical, behavioral, social, and health factors that are associated with accelerated aging among cancer survivors so that interventions can be developed and delivered effectively in a timely fashion. This need is especially critical for cancer survivors of less common cancers and non-white populations, for which the published literature is remarkably scant. Notably, it is important to determine whether certain factors associated with accelerated aging in the general population that are more prevalent among cancer survivors of marginalized identities (e.g., stress, discrimination, poverty) may interact with cancer treatment to further accelerated aging, leading to disparities in aging-related, and other downstream, adverse outcomes. Second, as this special section illustrates, there is a critical need for the development and large-scale testing of both pharmacologic and non-pharmacologic interventions to prevent, mitigate, and reverse accelerated aging in cancer survivor populations. Importantly, these interventions should be guided by principles of geroscience—i.e., the intervention should impact at least one hallmark of aging and, subsequently, aging-related disease outcomes [6]. Senolytics, which target cell senescence, are a promising pharmacologic intervention, but data do not yet exist on the efficacy of these drugs among cancer survivors.

Third, biomarkers linked to the underlying aging hallmarks and predict the development of aging phenotypes are an emerging area of interest and are urgently needed to identify survivors at risk of accelerated aging, guide treatment selection, and provide early intervention before the onset of chronic and late-emerging effects. Biomarkers can also lead to identifying novel therapeutic targets and developing non-therapeutic interventions that target aging biology. In addition, biomarkers could also be used to inform personalized medicine approaches with a targeted selection of therapy for patients with higher-risk toxicity profiles. While many aging biomarkers exist—including those related to cell senescence (e.g., p16), epigenetic changes (e.g., epigenetic clocks), telomere shortening (e.g., telomere length), and inflammation (e.g., IL-6, CRP) [15]—and are promising measures to be utilized in the above capacities in cancer survivor populations, there is no consensus as to which biomarkers are valid and reliable measures in the context of cancer and its treatment.

As the contributions in this special section indicate, much progress has been made in the area of cancer survivors and treatment-related accelerated aging over the past several decades, and, in particular, since the first NCI think tank in 2018. Continued progress will necessitate a multi-disciplinary approach, bringing together investigators from across the translational continuum with expertise that spans aging, cancer survivorship, data science, and other disease-specific foci. Such collaborations can help to fill critical gaps and bring us closer to a collective goal of improving cancer survivors’ quality of life and survival through evidence-based clinical care.

## References

[1] Siegel RL, Miller KD, Wagle NS, Jemal A. Cancer statistics, 2023. CA Cancer J Clin. 2023;73(1):17–48. 36633525 10.3322/caac.21763

[2] Miller KD, Nogueira L, Devasia T, et al. Cancer treatment and survivorship statistics, 2022. CA Cancer J Clin. 2022;72(5):409–36. 35736631 10.3322/caac.21731

[3] Bhatia R, Holtan S, Jurdi NE, et al. Do cancer and cancer treatments accelerate aging? Curr Oncol Rep. 2022;24(11):1401–12. 35796942 10.1007/s11912-022-01311-2 PMC9606015

[4] Henderson TO, Ness KK, Cohen HJ. Accelerated aging among cancer survivors: from pediatrics to geriatrics. Am Soc Clin Oncol Educ Book. 2014;34:e423-30. 10.14694/EdBook_AM.2014.34.e423 24857133

[5] Mohamed M, Ahmed M, Williams AM, et al. A scoping review evaluating physical and cognitive functional outcomes in cancer survivors treated with chemotherapy: charting progress since the NCI think tank on cancer and aging phenotypes. J Cancer Surviv. 2018. 10.1007/s11764-024-01589-0 PMC11324695 38743185

[6] Guida JL, Ahles TA, Belsky D, et al. Measuring aging and identifying aging phenotypes in cancer survivors. J Natl Cancer Inst. 2019;111(12):1245–54. 31321426 10.1093/jnci/djz136 PMC7962788

[7] National Cancer Institute. Measuring aging and identifying aging phenotypes in cancer survivors. Meeting summary from think tank #1. 2018. https://cancercontrol.cancer.gov/sites/default/files/2020-06/thinktank1_report_measuring-aging-and-identifying-aging-phenotypes-in-cancer-survivors.pdf. Accessed 1 Dec 2023

[8] Sedrak MS, Sun CL, Bae M, et al. Functional decline in older breast cancer survivors treated with and without chemotherapy and non-cancer controls: results from the Hurria Older PatiEnts (HOPE) prospective study. J Cancer Surviv. 2024. 10.1007/s11764-024-01594-3 PMC11324395 38678525

[9] Westrick AC, Zhu P, Friese C, et al. The association of pre-cancer diagnosis cardiovascular risk factors with memory aging after a cancer diagnosis, overall and by race/ethnicity. J Cancer Surviv. 2024. 10.1007/s11764-024-01593-4 PMC11465937 38647590

[10] Berkman AM, Goodenough CG, Durakiewicz P, et al. Associations between mitochondrial copy number, exercise capacity, and physiologic cost of walking and cardiac strain in young adult survivors of childhood cancer. J Cancer Surviv. 2024. 10.1007/s11764-024-01590-7 PMC11324404 38635100

[11] Rosko AE, Elsaid M, Woyach J, et al. Determining the relationship of p16INK4a and additional molecular markers of aging with clinical frailty in hematologic malignancy. J Cancer Surviv. 2024. 10.1007/s11764-024-01591-6 PMC11324703 38678524

[12] Hurria A, Togawa K, Mohile SG, et al. Predicting chemotherapy toxicity in older adults with cancer: a prospective multicenter study. J Clin Oncol. 2011;29(25):3457–65. 21810685 10.1200/JCO.2011.34.7625 PMC3624700

[13] Winters-Stone KM, Stoyles S, Dieckmann N, et al. Can strength training or tai ji quan training reduce frailty in postmenopausal women treated with chemotherapy? A secondary data analysis of the GET FIT trial. J Cancer Surviv. 2023. 10.1007/s11764-024-01592-5 38642204

[14] Guida JL, Agurs-Collins T, Ahles TA, et al. Strategies to prevent or remediate cancer and treatment-related aging. J Natl Cancer Inst. 2021;113(2):112–22. 32348501 10.1093/jnci/djaa060 PMC7850536

[15] Sedrak MS, Gilmore NJ, Carroll JE, et al. Measuring biologic resilience in older cancer survivors. J Clin Oncol. 2021;39(19):2079–89. 34043454 10.1200/JCO.21.00245 PMC8260901

